# METTL21B is a prognostic biomarker and potential therapeutic target in low-grade gliomas

**DOI:** 10.18632/aging.203454

**Published:** 2021-08-26

**Authors:** Xin Shu, Xinquan Li, Xiaochen Xiang, Qiang Wang, Qingming Wu

**Affiliations:** 1 Institute of Infection, Immunology and Tumor Microenvironment, Hubei Province Key Laboratory of Occupational Hazard Identification and Control, Medical College, Wuhan University of Science and Technology, Wuhan 430065, China

**Keywords:** eukaryotic translation elongation factor 1A (eEF1A), methyltransferase, METTL21B, low grade glioma, prognosis

## Abstract

A considerable amount of literature has demonstrated that eukaryotic translation elongation factor 1A (eEF1A) is closely related to tumors. As a newly identified lysine specific methyltransferase targeting eEF1A at Lys-165, too little attention has been paid to the function of METTL21B. To determine the potential significance and prognostic value of METTL21B in low grade glioma (LGG), we analyzed the expression, methylation level and copy number variations (CNV) of METTL21B and its effect on prognosis in patients with LGG by 4 public databases in conjunction with experimental examination of LGG patient samples. As a result, we found that high expression, hypomethylation and gain/amplification of CNV of METTL21B were associated with poor prognosis in LGG. The potential functions of METTL21B in LGG may be involved in cell adhesion, angiogenesis and cell proliferation of tumor by enrichment analysis. In addition, METTL21B may facilitate immune evasion of tumor and affect prognosis by mediating macrophage polarization from M1 to M2 and regulating expression of immune checkpoints. Nevertheless, patients with high METTL21B level are likely to have better response to immune checkpoints blockage therapy. Because of its substrate specificity, METTL21B is expected to be a promising target for the treatment of glioma.

## INTRODUCTION

Arising from glial cells, glioma is a type of primary neoplasms of center nervous system (CNS) system in adults, with the highest morbidity and mortality [1]. According to 2016 World Health Organization (WHO) classification standard of tumors of CNS, gliomas are divided into four grades (I, II, III, and IV), among which grades I, II and III are attributed as low-grade gliomas (LGGs) [2]. Although the patients with LGG had a longer survival time compared with glioblastoma (GBM, WHO grade IV), its high postoperative recurrence and mortality rates are still significant concerns [3]. More than half of LGG patients died of recurrence and progression [4]. Despite the developments of treatment for LGG in recent years, some tumors still showed therapeutic resistance ultimately and even progressed to GBM [5, 6]. Genetic heterogeneity of LGG is considered as the main reason of different treatment responses and widely variable prognosis [3, 7]. For example, a great quantity of studies demonstrated that IDH mutation increases the sensitivity of temozolomide chemotherapy and represents a favorable prognostic factor in LGG patients [8, 9]. In addition, some recent research also reported that MGMT promoter methylation, TERT promoter mutation, TLX1NB, ARL9 and 1p/19q deletion are association with prognosis of patients with LGG [10–14]. Taken together, identification of molecular biomarkers is unquestionably essential and urgent for accurately predicting prognosis and for seeking critical therapeutic targets in LGG.

Protein methylation is ubiquitous and acts as a pivotal regulator of various cellular biological processes in human and other organisms [15, 16]. Increasingly emerging evidence has found that histone and non-histone methylation are involved in cellular pathways associated with cancer [17–19]. METTL21B, also known as FAM119B or EEF1AKMT3, is a member of methyltransferase-like protein family which contain a seven-beta-strand methyltransferase domain and can regulate methylation modification of a variety of substrates including proteins and nucleic acids [20]. Dysregulation of METTL21B has been implicated in several human diseases and pathological processes, such as multiple sclerosis, rheumatoid arthritis and neurodegenerative disease [20–22]. Eukaryotic translation elongation factor 1A (eEF1A), as a subunit of eEF1 which promotes the delivery of aminoacyl-tRNA to the ribosome during translation, has been demonstrated as the only target substrate of METTL21B to the present date [23, 24]. Several recent studies have showed that METTL21B can catalyze the methylation of eEF1A on Lys-165 and impose a significant impact on translation of mRNAs [23, 24]. There is a tight relationship between dysregulation of eEF1A and cancer. For instance, overexpression of eEF1 is associated with poor prognosis of many types of cancers including breast, lung and liver cancer [25]. Besides, di-methylation of eEF1A lysine 55 (eEF1AK55me2) catalyzed by METTL13 can increase translational output and then promote tumorigenesis [26]. However, the prognostic significance and molecular function of METTL21B is still undetermined in glioma.

In our research, by using The Cancer Genome Atlas (TCGA), the Chinese Glioma Genome Atlas (CGGA), Rembrandt dataset and Gravendeel/GSE16011 dataset, we assessed the expression level (in conjunction with experimental examination of LGG patient samples), methylation level and copy-number variations of METTL21B in LGG tissues, analyzed the association between prognosis and expression of METTL21B, and explored the underlying molecular mechanisms of METTL21B in LGG. Furthermore, to increase the understanding of function of METTL21B in tumor immune microenvironment, we evaluated the effect of METTL21B on immune infiltration level and expression of immune checkpoints in LGG. Additionally, we also analyzed the correlation between METTL21B and four other genes which were also involved in the methylation regulation of eEF1A, and established the prognostic nomogram.

## MATERIALS AND METHODS

### Data mining

4 glioma public datasets comprising 1253 LGG patients (TCGA-LGG cohort: 509 patients; CGGA database: 443 patients; Rembrandt dataset: 184 patients and Gravendeel/GSE16011 dataset: 117 patients) were used for analysis [6, 27–29]. Firstly, the mRNA expression levels of METTL21B were compared between LGG tissues and normal samples by using the online website Gene Expression Profiling Interactive Analysis (GEPIA) (http://gepia.cancer-pku.cn/index.html), which included expression data from TCGA and GTEx database [30]. Besides, we downloaded transcriptome data of 337 samples from GSE16011 dataset (117 tumors and 8 normal tissues) and Rembrandt dataset (184 tumors and 28 normal tissues) to verify the expression differences of METTL21B. Then, the clinical information and transcriptional profiles were extracted from TCGA-LGG cohort and CGGA database for further analysis. The methylation level and copy-number variations (CNV) data of METTL21B were also downloaded via the Cbioportal Website. (https://www.cbioportal.org/) [31].

### Experimental examination of LGG patient samples

Between June 2011 and November 2014, a series of 31 low grade glioma samples were collected from patients who underwent surgery at the Department of Neurosurgery, Associated Hospital affiliated to Wuhan University of Science and Technology. Of them, 3 samples were WHO grade I (pilocytic astrocytoma), 13 cases were WHO grade II and 15 cases were WHO grade III. All patients were followed up after hospitalization with a mean duration of 75 months (range 36-122 months). The follow-up period was calculated as the duration from the date of surgery to death, or until April 2021 for surviving patients. Recurrence was defined as local tumor growth on the basis of clinical symptoms and neuroimaging findings. This study was approved by local ethical authorities of Medical school of Wuhan University of Science and Technology in accordance with the Helsinki Criteria (No. 202176). Written informed consent was obtained from each individual patient.

Briefly, samples were dissected into two halves with one half immediately frozen at -80° C for RNA extraction and the second half fixed with 10% formalin and embedded in paraffin for immunohistochemistry (IHC) staining. RNA isolation and qPCR were performed as described previously [32]. The primer sequences of human METTL21B and GAPDH were designed by GenePharma (Shanghai, China) (Supplementary Table 1). For IHC staining, Tissue sections were deparaffinized in xylene, rehydrated through an ethanol gradient and washed for 15 min with phosphate-buffered saline. For antigen retrieval, sections were heated in 0.01 M citrate buffer (pH 6.0) for 20 min in a microwave. Endogenous peroxidase activity was blocked by 3% hydrogen peroxidase in methanol for 10 min. Nonspecific antibody binding was blocked by incubation with the appropriate serum for 20 min which reduced positive signals in vascular, stromal and blood cells. Sections were incubated with primary antibody (HPA043020, 1:500, Sigma-Aldrich Co. Ltd, German) overnight at 4° C in a humidified chamber and were subsequently treated with biotinylated secondary antibodies against mouse IgGs for 30 min (ABC Elite, Vector Laboratories, Burlingame, CA) and for 30 min with avidin-biotin complex (ABC Elite), followed by treatment with 0.06% diaminobenzidine (Sigma Chemical, St. Louis, MO, USA) and 0.01% hydrogen peroxidase for 5 min. All sections were counterstained with hematoxylin. Antibody specificity control stains were prepared by omitting primary antibodies. The percentage of cells stained positive compared to the total number of tumor cells was determined using Image J software (NIH, Bethesda, MD, USA) by counting around 400 tumor cells at 400X magnification.

### Prognosis analysis

According to the expression values of METTL21B, patients from our clinical department and those from the employed database were independently split into two groups (low vs. high expression group) based on the median value. Survival analysis was performed by Kaplan-Meier method to analyze the effect of expression, methylation and CNV of METTL21B on prognosis of LGG patients. The timeROC curve was plotted to evaluate the accuracy of METTL21B for predicting prognosis of patients with LGG by timeROC package of R software. In addition, multivariable cox regression analysis was employed to adjust for clinicopathological features including age, gender, tumor grade, IDH mutation status and radiotherapy.

### DEGs, KEGG pathways, GO analysis and gene sets enrichment analysis (GSEA)

In TCGA-LGG database, differential expression genes (DEGs) between low and high METTL21B expression group were identified by Limma package of R software. Log_2_|fold change|>1 and adjusted P < 0.05 were defined as the thresholds for DEGs screening. Functional enrichment analyses including Gene Ontology (GO) and Kyoto Encyclopedia of Genes and Genomes (KEGG) enrichment analysis of DEGs were performed to better understand the underlying function and carcinogenesis of METTL21B in LGG. KEGG pathway analysis was carried out by ClusterProfiler package of R software, and GO enrichment was analyzed by Metascape database. In additional, we utilized the MSigDb Hallmark gene set to execute the gene sets enrichment analysis by GSEA software (v4.1.0).

### Analysis of correlation between METTL21B and immune signature

Tumor Immune Estimation Resource (TIMER) (https://cistrome.shinyapps.io/timer/) is a web server which can be used to evaluate levels of immune infiltration and to analyze the association between immune infiltration and prognosis [33]. The ESTIMATE immune score of each sample was calculated by applying Estimate package of R software. Furthermore, the CIBERSORT algorithm was used to assess composition of 22 immune cells in tumor microenvironment by CIBERSORT package.

SIGLEC15, TIGIT, PDCD1LG2, PDCD1, CD274, LAG3, HAVCR2 and CTLA4 were selected to be immune checkpoint genes [34, 35], and the association between expression levels of these genes and METTL21B was analyzed in LGG. Tumor mutation burden (TMB) was computed by Maftools R package.

### Construction of prognostic model of eEF1A associated methyltransferases

Except for METTL21B, EEF1AKMT1(N6AMT2), EEF1AKMT2(METTL10), EEF1AKNMT(METTL13) and EEF1AKMT4(ECE2) are also methyltransferases involved in methylation regulation of eEF1A [36]. The association between expression of these genes and METTL21B was analyzed, and nomogram was constructed by ‘rms’ R package.

### Statistical analysis

Survival analysis and multivariate cox analysis were performed by “survival” and “survminer” package. Quantitative variables were compared by Wilcox-test, Student’s t-test or ANOVA with TukeyHSD test. Correlation analyses were assessed by using the Spearman or Pearson’s correlation test. R software (v4.0.2) was used for statistical analysis and graphing. P < 0.05 indicates statistical significance of difference.

### Availability of data and materials

The datasets used and/or analyzed during the current study are available from the corresponding author on reasonable request.

## RESULTS

### Expression level of METTL21B in LGG

**Figure 1:**
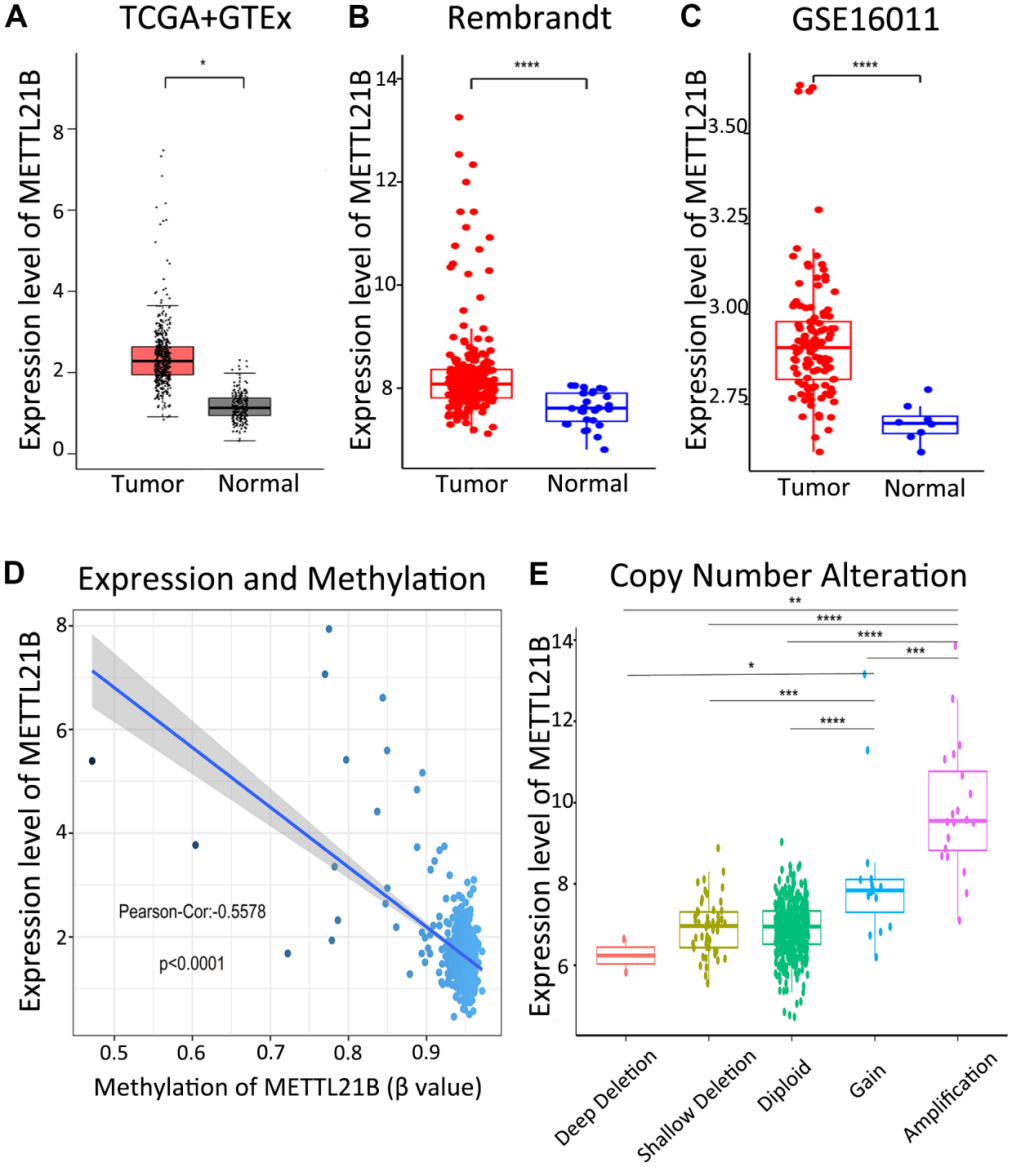
**Expression of METTL21B in LGG.** The expression differences of METTL21B between LGG tissues and normal samples in TCGA+GTEx (**A**), Rembrandt (**B**) and GSE16011 (**C**) dataset; (**D**) The correlation between expression and methylation of METTL21B. (**E**) Copy number gain/amplification of METTL21B markedly increased the mRNA expression. *P<0.05; **P<0.01; ***P<0.001; ****P<0.0001.

We determined the difference in expression of METTL21B between tumor tissues and normal tissues for 33 types of human cancers from TCGA and GTEx database by the GEPIA website (Supplementary Figure 1A). The data indicated that mRNA expression of METTL21B in LGG was significantly up-regulated compared with normal tissues (Figure 1A). We further verified that METTL21B is highly expressed in LGG tissues by Rembrandt database (Figure 1B) and GSE16011 dataset (Figure 1C).

As shown in Figure 1D, a significantly negative correlation (Pearson-Cor = −0.5578, P < 0.0001) was observed between mRNA expression level and DNA methylation of METTL21B. Besides, we explored the effect of copy number variation on METTL21B expression and showed that the copy number gain/amplification of METTL21B markedly increased the mRNA expression, while deletion of METTL21B copy number had no effect on its mRNA expression. (Figure 1E).

**Figure 2:**
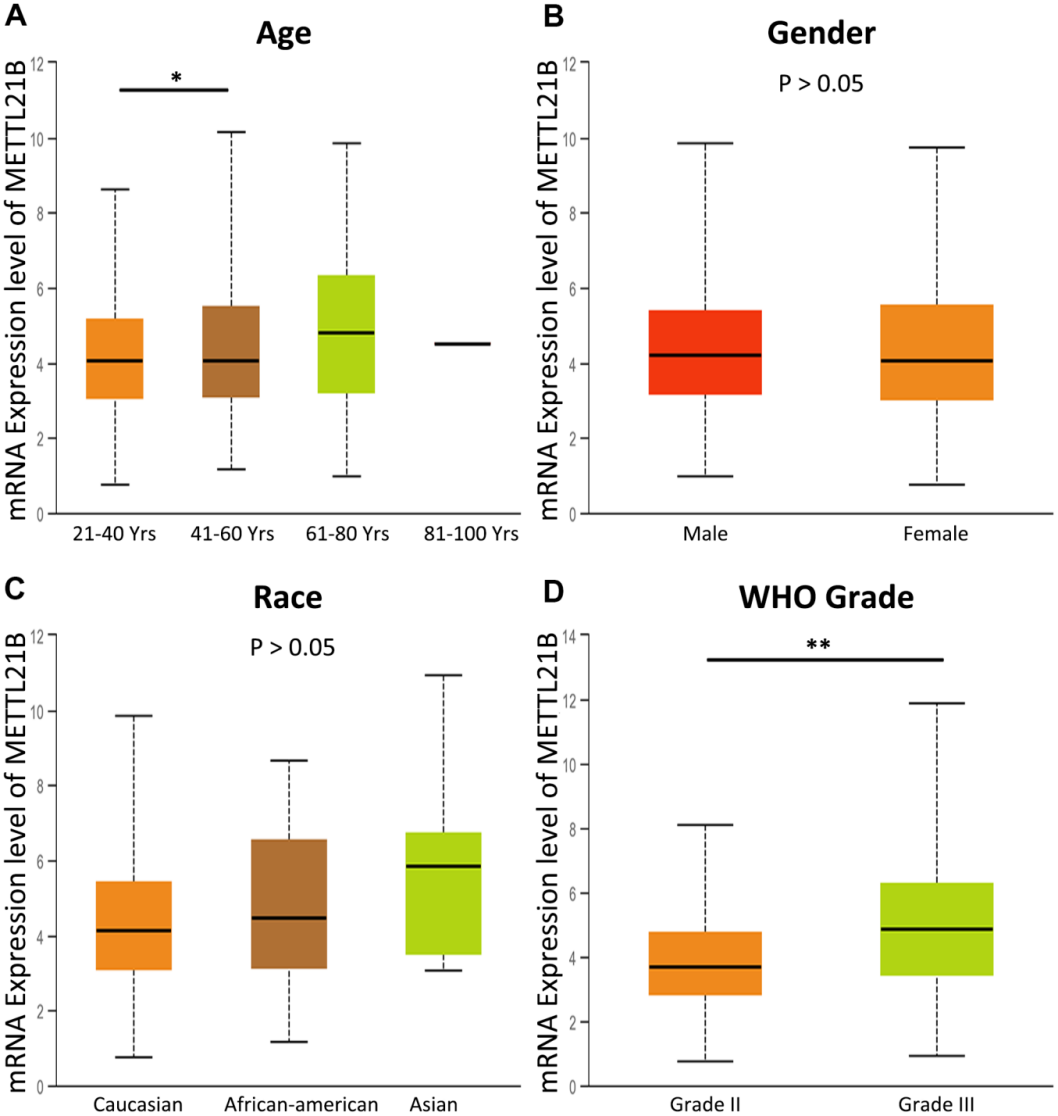
**The association between METTL21B expression and clinical features.** (**A**) age; (**B**) gender; (**C**) race; (**D**) WHO grade. *P<0.05; **P<0.01.

By analyzing the association between METTL21B expression and clinical features including age, gender, race and WHO grade, we found that WHO grade III glioma has higher expression level of METTL21B compared with WHO grade II (Figure 2), which indicates that METTL21B could be related to malignant behaviors of LGG.

**Figure 3:**
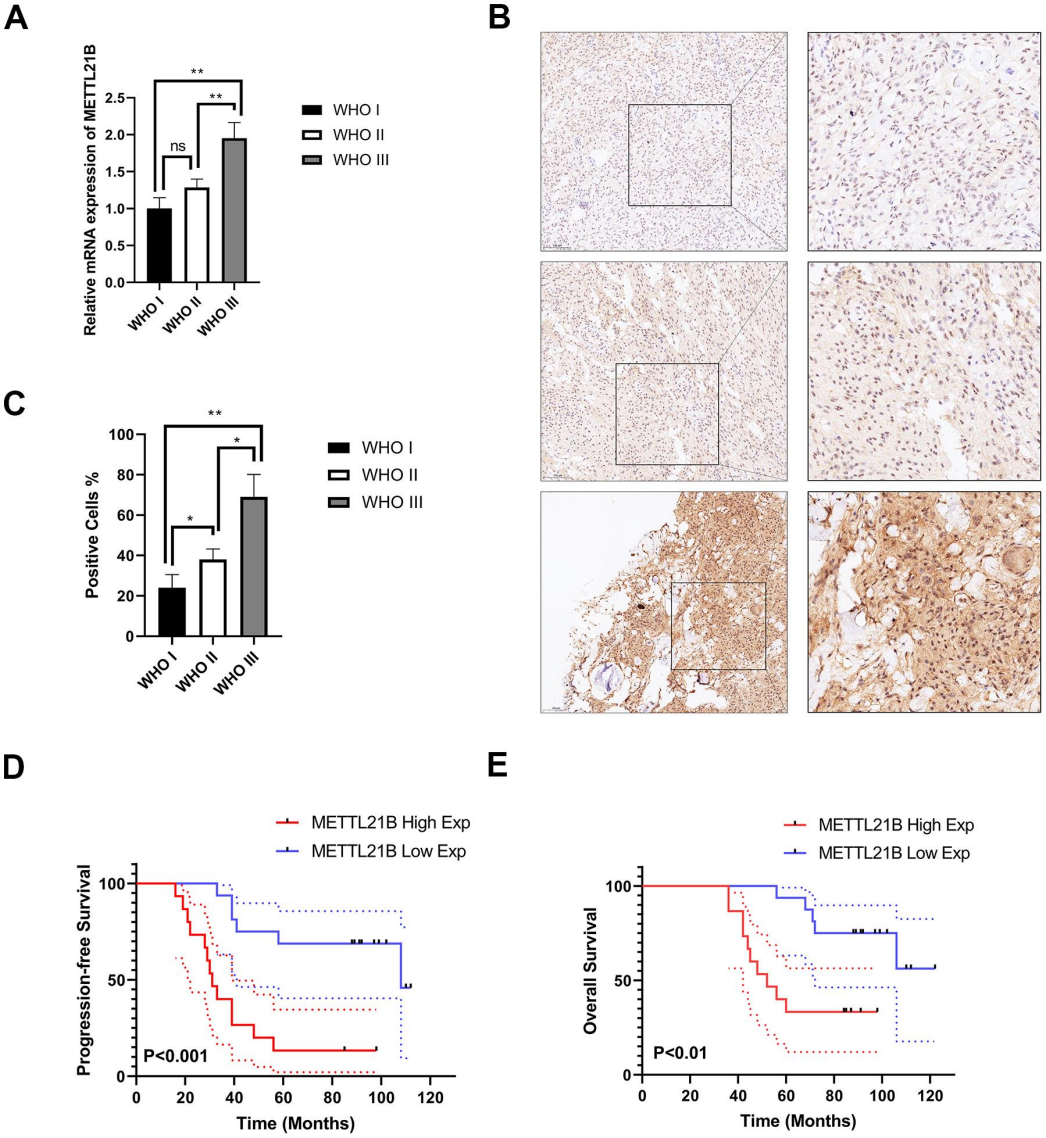
**Experimental examination of METTL21B expression and prognosis analysis in LGG patient clinical samples.** (**A**) The relative mRNA expression level in LGG with different WHO grades. (**B**) Representative immunohistochemistry images for METTL21B in LGG with different WHO grades. Positive cells showed nuclear and/or cytoplasmic brown staining while negative cells showed blue nuclei counter staining. (**C**) METTL21B positive cells were counted in LGG with different WHO grades. *P<0.05; **P<0.01; ns: no significance. (**D**, **E**) METTL21B high expression predicts a worse progression-free and overall survival (P<0.001 and P<0.01, respectively).

We further verified the expression of METTL21B in LGG by examining a series of 31 clinical specimens. As a result, there was a significantly higher mRNA expression level in WHO grade III than in WHO grade I/II (Figure 3A). In addition, immunohistochemistry (IHC) staining confirmed that the expression of METTL21B was positively associated with WHO grade at the protein level (Figure 3B, 3C). These results were in accordance with those from databases.

### Prognostic value of METTL21B in LGG

**Figure 4:**
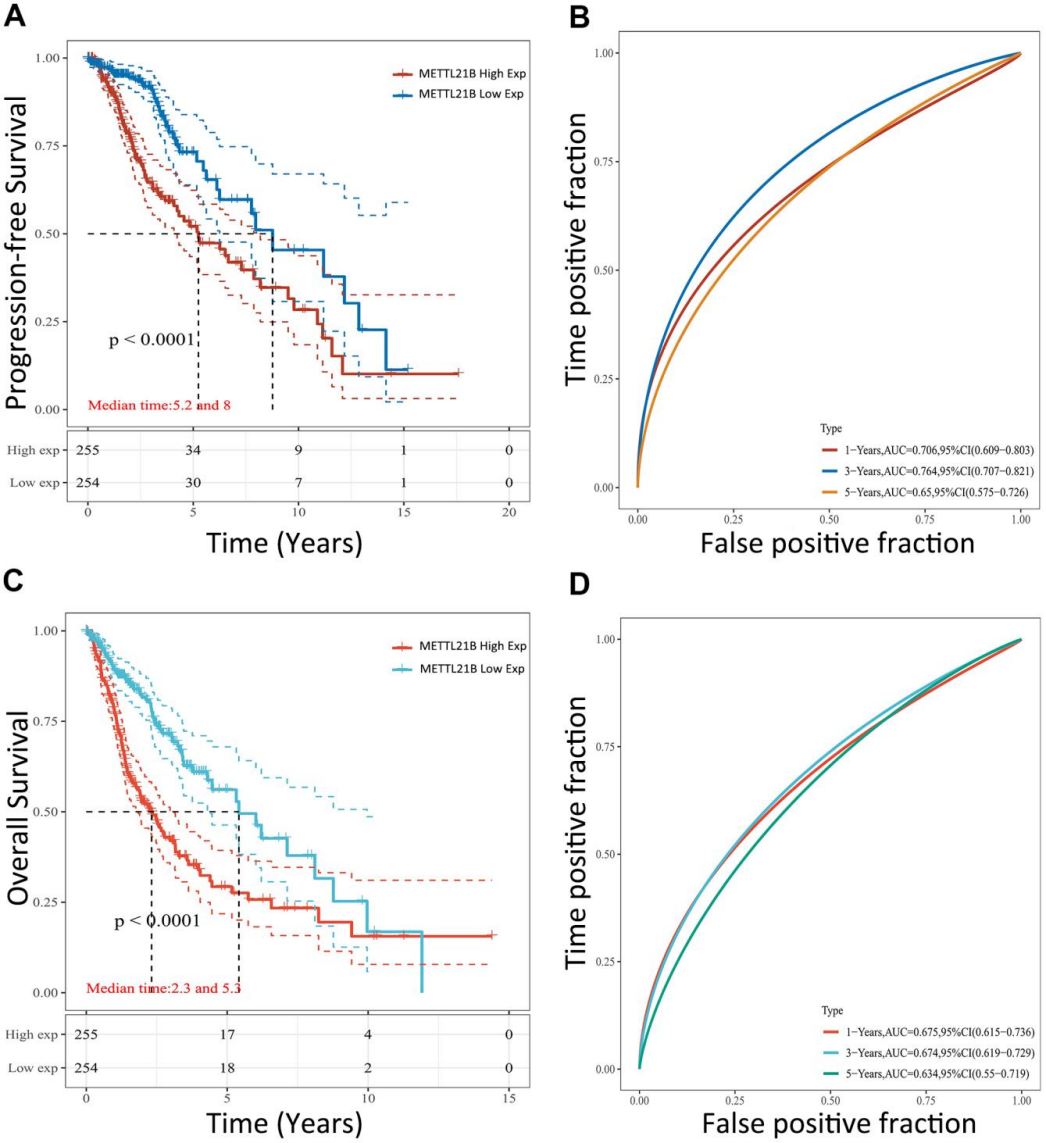
**Prognostic value of METTL21B for patients with LGG in TCGA.** The effect of METTL21B expression on overall survival (**A**) and progression-free survival (**C**) of patients; The ROC curve for OS (**B**) and PFS (**D**).

Survival heat map in Supplementary Figure 1B showed the correlation of METTL21B expression and prognosis of 33 cancer types in TCGA. Although the expression of METTL21B was remarkably up-regulated or down-regulated in 7 types of cancers compared with normal tissues as described in Supplementary Figure 1A, the prognosis was only associated with METTL21B expression in LGG. As exhibited in Figures 3D, 3E, 4, Kaplan-Meier curves were plotted to evaluate the effect of METTL21B on overall survival (OS) and progression-free survival (PFS) of patients in our clinical specimens and TCGA-LGG cohort. Patients with higher METTL21B expression had significantly poorer overall survival (median OS: 5.2 vs. 8.0 years, log-rank p<0.0001) and progression-free survival (median PFS: 2.3 vs. 5.3 months, log-rank p<0.0001) (Figure 4A, 4C). The areas under the time-dependent ROC curves (AUC) for 1-year, 3-year and 5-year overall survival rates were 0.706, 0.764 and 0.65 respectively, which suggested METTL21B had favorable prognostic accuracy in LGG patients (Figure 4B). Besides, the poor prognosis in the group with high METTL21B expression was also validated by data from CGGA database (Supplementary Figure 2A) and Rembrandt database (Supplementary Figure 2B).

As expected, low methylation level of METTL21B was associated with poor prognosis (median OS: 5.6 vs. 9.8 years) among patients with LGG (Supplementary Figure 3A). In addition, median overall survival was shorter for patients with gain/amplification of METTL21B copy number (median OS: 2.5 vs. 7.9 years) (Supplementary Figure 3B).

Multivariate Cox proportional-hazards model analysis revealed that expression of METTL21B was still significantly correlated with OS (HR=2.34, P<0.0001) after adjusting age, gender, WHO grade, IDH-mutation status and radiotherapy, demonstrating that the gene is an independent prognostic factor in LGG (Table 1).

**Table 1:** Multivariate Cox model for infiltration of clinical features and METTL21B expression in LGG.

**Clinical characteristics**	**Progression-free survival**			**Overall survival**	
	**HR (95%CI)**	**P value**		**HR (95%CI)**	**P value**
Age	1.02(1.01-1.04)	0.0003*		1.05(1.03-1.07)	<0.0001*
Gender	0.72(0.52-0.99)	0.0444*		0.96(0.63-1.46)	0.8478
WHO grade	1.39(00.97-1.99)	0.0721		2.44(1.50-3.96)	0.0003*
IDH-mutation	1.00(0.69-1.46)	0.9898		0.90(0.53-1.53)	0.6919
Radiation therapy	0.84(0.58-1.23)	0.3754		1.25(0.71-2.20)	0.4378
METTL21B expression	2.34(1.67-3.30)	<0.0001*		2.01(1.26-3.20)	0.0034*

### Identification of DEGs between low and high METTL21B group and KEGG and GO analysis

**Figure 5:**
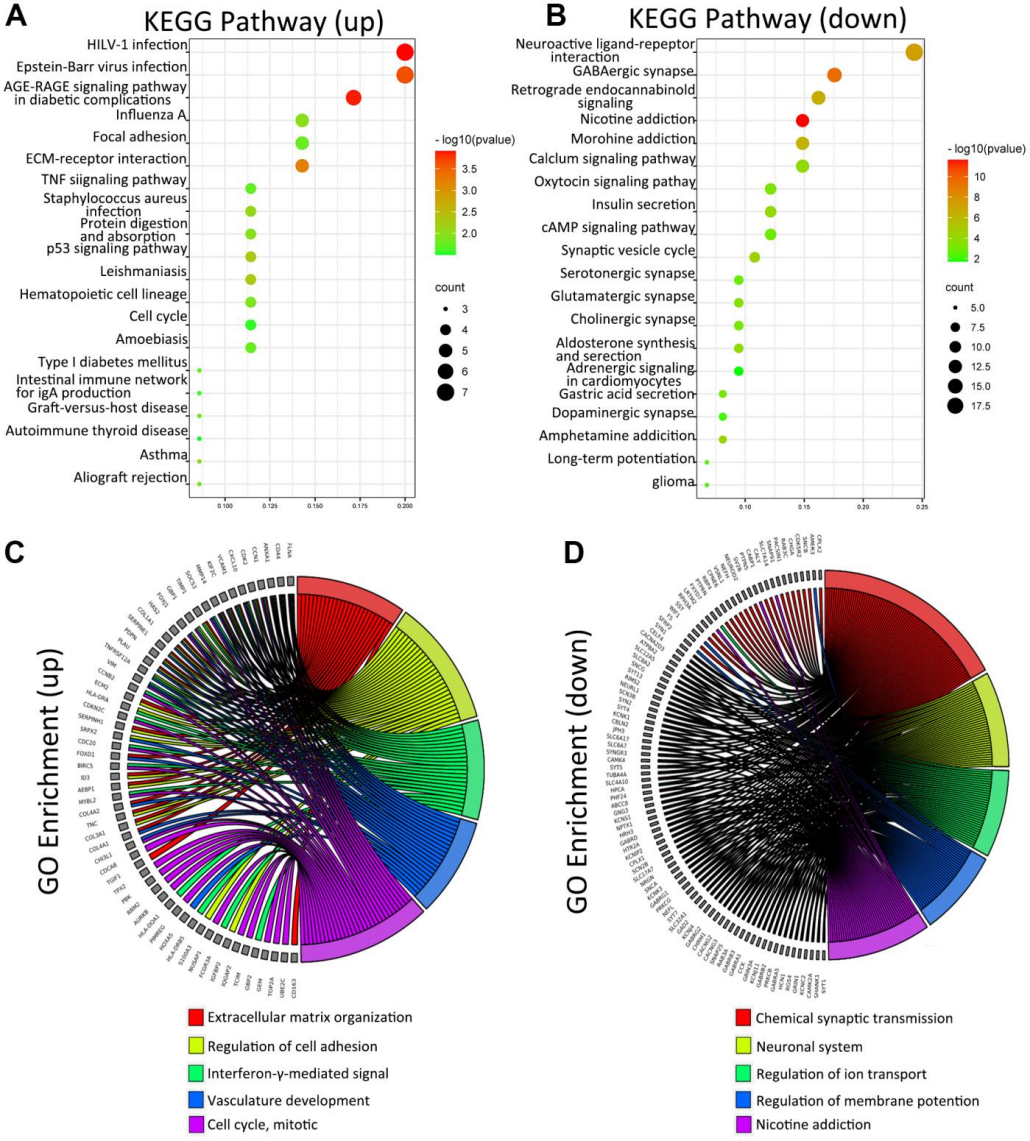
**KEGG and GO analysis of DEGs between low and high METTL21B expression groups.** KEGG analysis of DEGs (**A**, **B**); GO analysis of DEGs (**C**, **D**).

To understand the biological function of METTL21B in LGG, differential expression genes (DEGs) analysis was run between low and high METTL21B expression group by Limma package of R software. As depicted by the heatmap and volcano plot (Supplementary Figure 4A, 4B), 71 up-regulated genes (red dots) and 185 down-regulated genes (blue dots) were identified in high METTL21B expression group. KEGG enrichment analysis of DEGs showed that these up-regulated genes are mainly involved in Human T-cell leukemia virus 1 infection, Epstein-Barr virus infection, AGE-RAGE signaling pathway, ECM-receptor interaction and focal adhesion (Figure 5A, 5B). Additionally, the chord chart displayed 5 most enriched GO terms by different colors in high METTL21B expression group, including extracellular matrix organization, regulation of cell adhesion, interferon-gamma-mediated signal, vasculature development and cell cycle (Figure 5C, 5D). The results imply that METTL21B may be associated with cell adhesion, tumor immune, angiogenesis and cell proliferation of low-grade glioma.

### Results of gene set enrichment analysis

**Figure 6:**
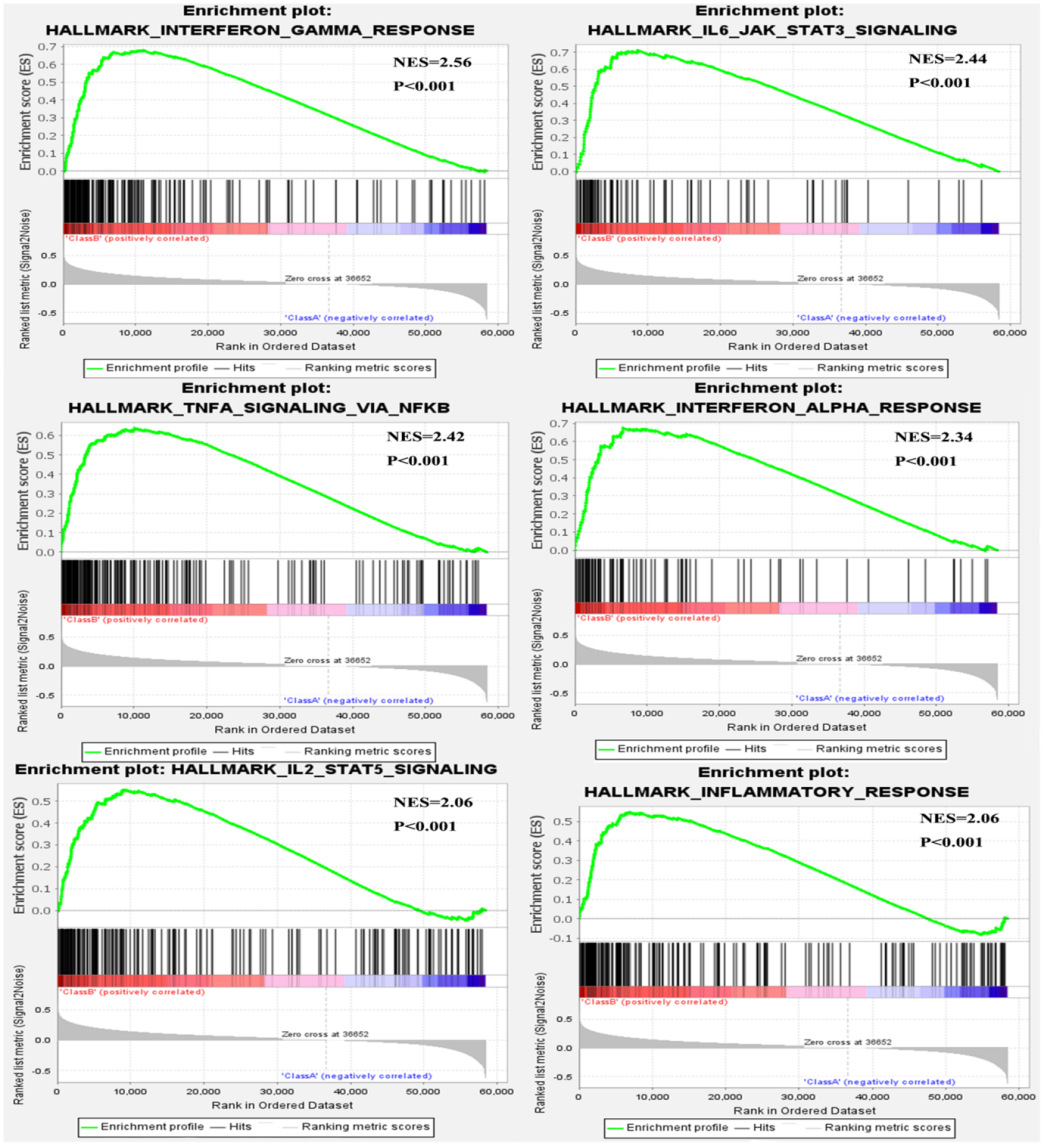
Six immune-related gene sets were enriched by GSEA.

We performed single gene GSEA to explore the METTL21B-related pathways in LGG. 27 out of 50 hallmark gene sets were significantly enriched in high METTL21B expression group at nominal p < 0.01 and FDR < 0.25, and of which 14 gene sets with normalized enrichment score (NES) > 2.0 were listed in Table 2. Among these gene sets, 2 gene sets are targets of E2F and MYC transcription factor families. “G2M_CHECKPOINT”, “EPITHELIAL_MESENCHYMAL_TRANSITION”, “ANGIOGENESIS”, “DNA_REPAIR” and “APOPTOSIS” are involved in tumorigenesis and tumor metastasis. Besides, 6 immune-related gene sets, including “INTERFERON_GAMMA_RESPONSE”, “IL6_JAK_STAT3_SIGNALING”, “TNFA_SIGNALING_VIA_NFKB”, “INTERFERON_ALPHA_RESPONSE”, “IL2_STAT5_SIGNALING” and “INFLAMMATORY_RESPONSE”, were also clearly up-regulated in the group with high METTL21B expression (Figure 6).

**Table 2:** 14 Gene sets with normalized enrichment score (NES) > 2.0 by GSEA.

**Gene set name**	**Size**	**NES**	**NOM p-val**	**FDR q-val**
HALLMARK_E2F_TARGETS	195	2.799	0	0
HALLMARK_G2M_CHECKPOINT	190	2.677	0	0
HALLMARK_INTERFERON_GAMMA_RESPONSE	198	2.560	0	0
HALLMARK_ALLOGRAFT_REJECTION	195	2.463	0	0
HALLMARK_EPITHELIAL_MESENCHYMAL_TRANSITION	197	2.442	0	0
HALLMARK_IL6_JAK_STAT3_SIGNALING	87	2.439	0	0
HALLMARK_TNFA_SIGNALING_VIA_NFKB	198	2.418	0	0
HALLMARK_INTERFERON_ALPHA_RESPONSE	95	2.336	0	0
HALLMARK_ANGIOGENESIS	36	2.291	0	0
HALLMARK_IL2_STAT5_SIGNALING	195	2.064	0	0
HALLMARK_INFLAMMATORY_RESPONSE	198	2.063	0	0
HALLMARK_DNA_REPAIR	148	2.039	0	0
HALLMARK_APOPTOSIS	159	2.029	0	0
HALLMARK_MYC_TARGETS_V1	194	2.013	0	0

### Association between METTL21B and immune infiltration

**Figure 7:**
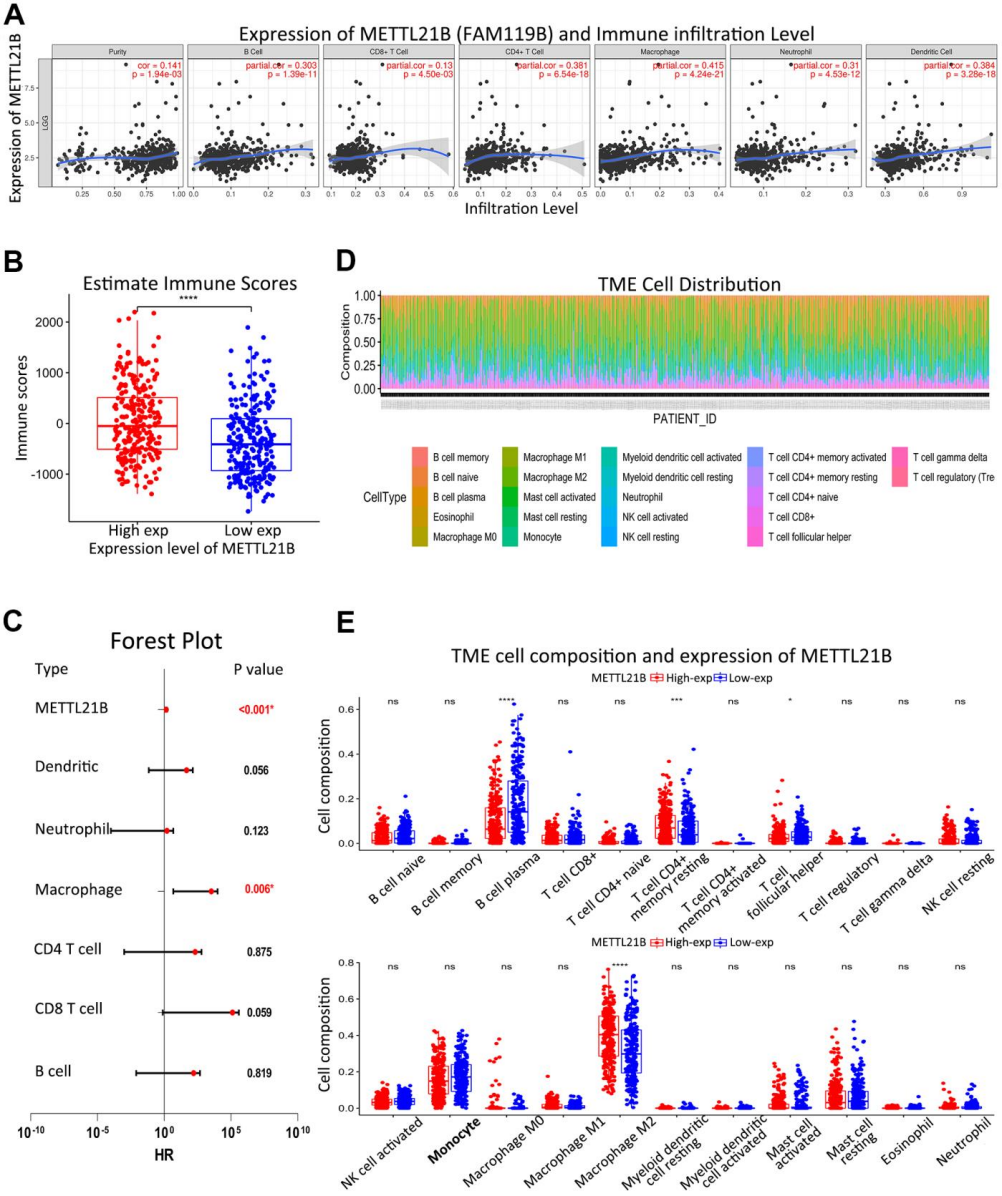
**Association between METTL21B and immune infiltration.** (**A**) Infiltration levels of all six immune cell types were significantly positively correlated with expression of METTL21B in LGG by TIMER. (**B**) Immune scores between low and high METTL21B expression group. (**C**) Multivariate Cox model for infiltration of six immune cell types and METTL21B expression. (**D**) Distribution of 22 subtypes of immune cells in all TCGA-LGG samples. (**E**) Infiltration levels of 22 subtypes of immune cells between low and high METTL21B expression group in LGG. *P<0.05; **P<0.01; ***P<0.001; ****P<0.0001; ns: no significance.

Due to the enrichment in several immune-associated signaling pathways in high METTL21B group, we investigated the association between METTL21B and immune microenvironment. The correlation between expression of METTL21B and infiltration levels of CD4+ T cells, CD8+ T cells, B cells, neutrophils, macrophage and dendritic cells was examined by TIMER website. As displayed in Figure 7A, infiltration levels of all six immune cell types were significantly positively correlated with expression of METTL21B in LGG after adjusting for tumor purity. Analogously, higher immune scores which were calculated by ESTIMATE algorithm based on transcription profile were observed in the group with up-regulated METTL21B (Figure 7B). Besides, macrophages displayed the strongest positive correlation with METTL21B expression level (r=0.415, p<0.001), and its high level of infiltration portended unfavorable prognosis for LGG patients (Figure 7C).

We further evaluated composition of 22 subtypes of immune cells in all TCGA-LGG samples by CIBERSORT method (Figure 7D). Higher percentage of M2 macrophage and resting memory CD4+ T cell were noticed in high METTL21B group, while infiltration levels of B cell plasma and T cell follicular helper were low (Figure 7E).

### METTL21B is associated with immune checkpoints and tumor mutation burden

**Figure 8:**
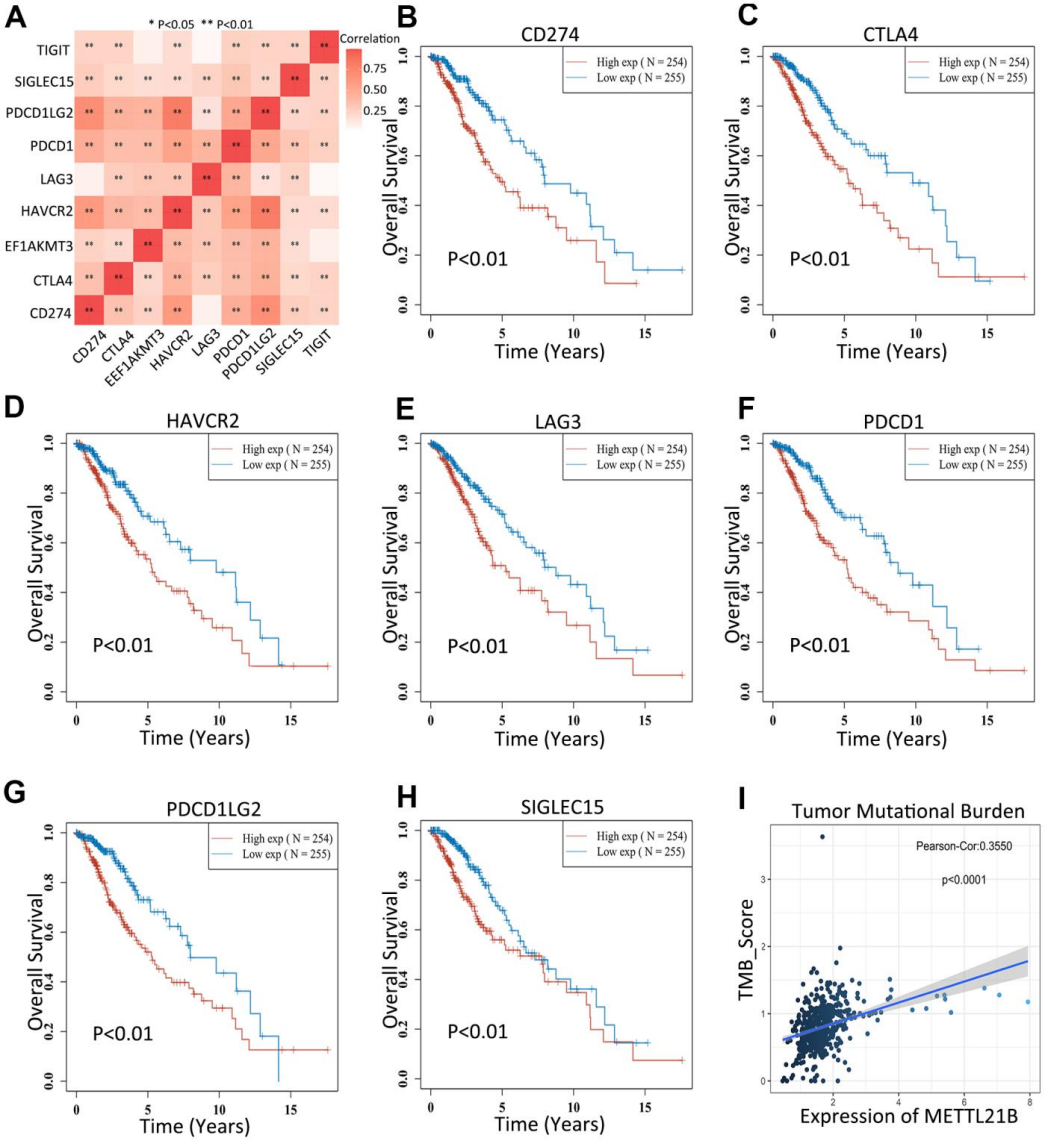
**METTL21B is correlated with immune checkpoints and tumor mutation burden.** (**A**) Correlation between expression of 8 immune checkpoints and METTL21B. The effects of CD274 (**B**), CTLA4 (**C**), HAVCR2 (**D**), LAG3 (**E**), PDCD1 (**F**), PDCD1LG2 (**G**) and SIGLEC15 (**H**) on prognosis of patients with LGG. (**I**) The correlation between expression of METTL21B and tumor mutation burden in LGG.

Increased expression of 7 immune checkpoints (CD274, CTLA4, HAVCR2, LAG3, PDCD1, PDCD1LG2 and SIGLEC15) were found in high METTL21B expression group (Supplementary Figure 5). Similarly, a significant positive correlation between expression of these immune checkpoints and METTL21B was confirmed by Spearman correlation analysis (Figure 8A). Of note, overexpression of all these 7 genes were associated with low OS in patients with LGG (Figure 8B–8H), which indicated METTL21B could be involved tumor immune escape and promote progression by regulating expression of immune checkpoints. Besides, the tumor mutation burden of LGG was markedly positively correlated with METTL21B, implying that patients with high expression level of METTL21B may be more likely to have favorable response to immunotherapy targeting immune checkpoints (Figure 8I).

### Construction of prognostic model of METTL21B along with other eEF1A associated methyltransferases

**Figure 9:**
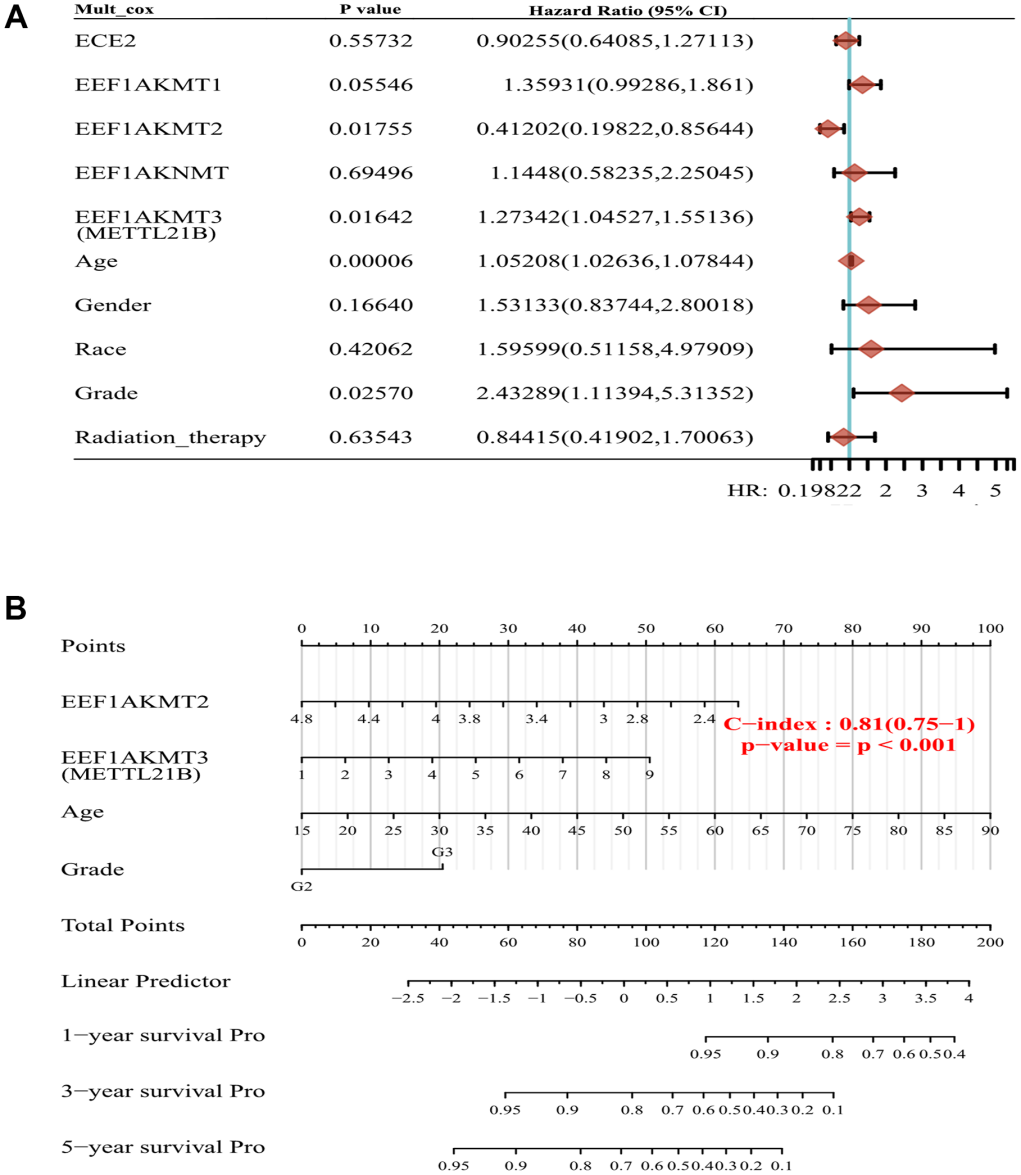
**Construction of prognostic model of METTL21B along with other eEF1A associated methyltransferases.** (**A**) Multivariate Cox model for 5 eEF1A associated methyltransferases and clinical features. (**B**) The nomogram for METTL21B, EEF1AKMT2, age and WHO grade.

EEF1AKMT1 (N6AMT2), EEF1AKMT2 (METTL10), EEF1AKNMT (METTL13) and EEF1AKMT4 (ECE2) are also regulatory factors of methylation modification of eEF1A. Taking this into consideration, we established a prognostic model of METTL21B along with other eEF1A associated methyltransferases in LGG. Multivariate Cox regression analysis found that age, WHO grade, METTL21B and EEF1AKMT2 were independent prognostic factors in LGG (Figure 9A).

Next, nomogram was plotted to visualize the model, by which the total points can be obtained to evaluate survival possibility of each patient (Figure 9B). C-index of the model is 0.81, which indicated the excellent predictive ability.

## DISCUSSION

As a newly identified lysine specific methyltransferase targeting eEF1A at Lys-165, too little attention has been paid to the function of METTL21B and the precise role of METTL21B in LGG are still unclear. In this study, we provide the first insights into the clinical significance and biological function of METTL21B in LGG by performing a series of in-depth bioinformatics analysis in conjunction with experimental examination of LGG patient samples. By the transcriptome analysis of nearly 1000 tumors and normal samples from 4 databases, we found that METTL21B mRNA levels are significantly higher in LGG compared with normal tissues and are associated with methylation and CNV of METTL21B. Besides, high METTL21B expression, METL21B hypomethylation and gain/amplification of copy number were found as poor prognostic indicators in TCGA-LGG cohort. Furthermore, the prognostic value of METTL21B expression was also validated in 2 other datasets. Altogether, these findings underlined that METTL21B could be a novel diagnostic and prognostic biomarker in LGG patients.

Function of protein is affected by its methylation modification, which could suggest that methylation of eEF1A by METTL21B have downstream consequences for cellular processes that eEF1A is involved in. In fact, a recent study has unraveled that alterations of METTL21B expression leads to substantial alterations in translation through dynamic regulation of methylation of eEF1A in mammalian cells [23]. Besides its canonical role in translation elongation, eEF1A is also involved in many other cellular activities, such as nuclear export, protein degradation, regulation of the cytoskeleton, apoptosis and so on [37, 38]. eEF1A also plays a vital role in carcinogenesis in multiple cancer types [25]. Additionally, methylation modification of eEF1A at lysine 55 by METTL13 was shown to be directly involved in tumorigenesis [26]. However, the mechanism underlying how METTL21B regulate malignant behaviors of LGG by methylation modification of eEF1A remains elusive. By KEGG and GO analysis of DEGs and gene set enrichment analysis, we identified some possible pathways and cellular processed associated with metastasis, tumor immune, angiogenesis and cell proliferation of tumor as above.

We also discovered that gene targets of MYC family and E2F family were enrichen in high METTL21B group. The E2F-family proteins can induce distinct cell cycle factors to regulate cell proliferation in U343 astrocytoma cells [39]. Besides, MYC also exerts an important effect on the promotion of mitosis in glioma [40]. These results indicate that METTL21B participated in the development of LGG by regulating activity of E2F and MYC transcription factors families.

Accumulating evidence suggests that the tumor immune microenvironment is known to be tightly associated with tumor progression and prognosis of patients [41]. Our results unveiled that METTL21B is significantly positively correlated with infiltration levels of a variety of immune cells, suggesting METTL21B could have a profound influence on the tumor immune microenvironment in LGG. Particularly, macrophage infiltration showed the strongest correlation with METTL21B expression level (r=0.415, p<0.001), and was associated with unfavorable prognosis in LGG patients. Furthermore, compared with low METTL21B group, percentage of M2 macrophage is higher in the group with high METTL21B expression. Unlike anti-tumor function of M1 phenotype, M2 macrophage is deemed immunosuppressive and pro-tumorigenic [42]. Many studies have reported that high levels of TGF-β, EGF, MMP-2, MMP-9 and IL-10, and low levels of IL-12 can be produced by M2 polarized glioma-associated microglia/macrophages to promotes invasion, proliferation, immune evasion and angiogenesis of glioma [43]. In low-grade glioma, the number of CD68+ (a marker of M2) cells is positively associated with malignancy degree and is inversely related to the recurrence-free survival [44, 45]. Herein, we speculate that METTL21B may facilitate immune evasion of tumor and worsen prognosis by mediating macrophage polarization from M1 to M2.

A recent study has showed that eEF1A2, a subtype of eEF1A, can regulate expression and release of some cytokines in brain tumors, which implied EEF1A is associated with tumor immune microenvironment [46]. IL-8 was involved in macrophage polarization from M1 to M2 in glioma, and was founded to be up-regulated in EEF1A2-overexpressed U87-MG cell line [46, 47]. On the other hand, knockdown of EEF1A2 decreased the expression of IL-6 which is considered to play key roles in tumor immune evasion [46, 48, 49]. Thus, METTL21B may regulate tumor immune microenvironment by eEF1A.

As surface membrane receptors of immune cells, immune checkpoints are used by tumors to escape immune surveillance [50]. As mentioned above, patients with high METTL21B expression levels accompanied up-regulation of multiple immune checkpoints, implying a significant role of METTL21B in immune suppression of tumor. On the other hand, patients with high immune checkpoints expression were more likely to benefit from immune checkpoint inhibitors-based immunotherapy [51, 52]. Besides, tumor mutational burden (TMB) has become a potential biomarker and can be utilized to predict immune checkpoint inhibitors efficacy in a host of cancer types [53, 54]. A higher TMB usually results in more neoantigens, thereby increasing immunogenicity of tumor and antitumor activity [53]. Based on this, it is reasonable to assume that patients with high METTL21B level may have better immunotherapeutic response, which is beneficial for designing and implementing suitable individualized treatment regimens to improve patients' long-term prognosis.

Except for METTL21B, 4 other methyltransferases have been discovered to be involved in methylation regulation of eEF1A at different sites until now, and eEF1A is also expected to be their only target protein for most of them [36]. Specific methylation sites have distinct functions in modulating eEF1A. For example, upregulation of ribosomal proteins and alternations of some eEF1A-associated cellular processes were detected in METTL21B-knockout cells compared with EEF1AKMT1-knockout cells by proteomic analysis [24]. In our results, interestingly, EEF1AKMT2 was recognized as a favorable prognostic factor. The finding suggested that functions of eEF1A may be completely opposite when it was methylated at different sites in LGG. Hence, it is tempting to design targeted drugs for the treatment of glioma due to the specificity of METTL21B.

Collectively, the present study confirmed that METTL21B is a promising prognostic biomarker and therapeutic target in LGG for the first time by bioinformatics analysis and experimental validation. Nevertheless, there are some unavoidable limitations in our study. Firstly, the effect of methylation and CNV of METTL21B on prognosis were unable to be validated in other dataset because of the lack of data. Besides, we can’t obtain the methylation data of eEF1A in LGG from public databases. Thus, the associated between methylation level of eEF1A and METTL21B expression couldn’t be analyzed in present study. Finally, further *in vitro* and *in vivo* molecular biological experiments are needed be performed to demonstrate concrete mechanisms by which METTL21B regulates malignant behaviors of LGG, especially the correlation with eEF1A and tumor immune microenvironment.

## Supplementary Material

Supplementary Figures

Supplementary Table 1
