# Supplementary material for: METTL21B is a prognostic biomarker and potential therapeutic target in low-grade gliomas
Source: Aging (Albany NY). 2021 Aug 26;13(16):20661–83. doi: 10.18632/aging.203454 (PMC8436898; doi:10.18632/aging.203454)
Supplement: Supplementary Figures [file aging-13-203454-s001.pdf]

SUPPLEMENTARY FIGURES

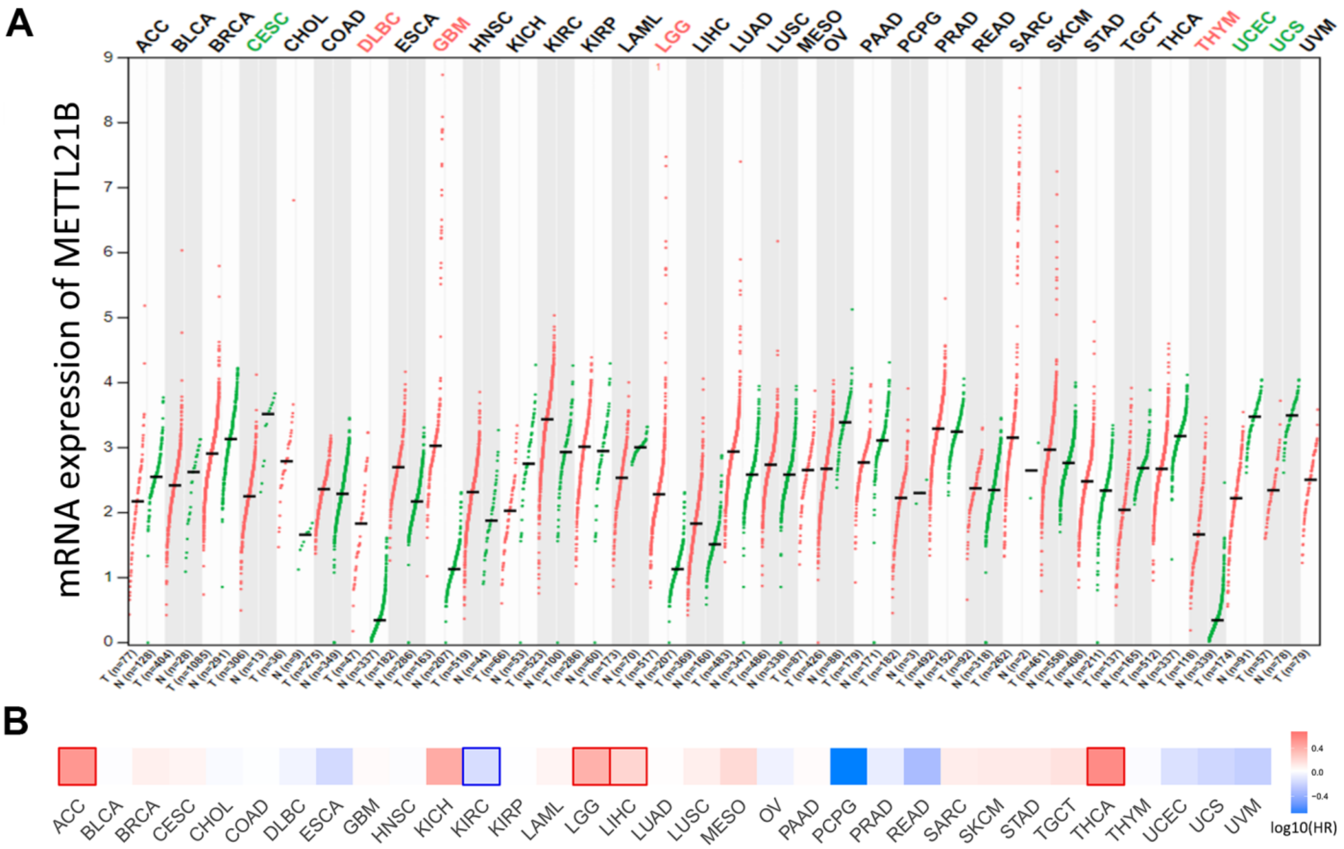

Supplementary Figure 1. Pan-cancer analysis of METTL21B expression among 33 types of cancers from TCGA. (**A**) The difference in expression of METTL21B between tumor tissues and normal tissues of 33 types of human cancers from TCGA and GTEx database. Red/Green fonts indicate statistically significant differences. (**B**) Survival heat map of 33 cancer types in TCGA. Red/Blue border lines indicate statistically significant differences.

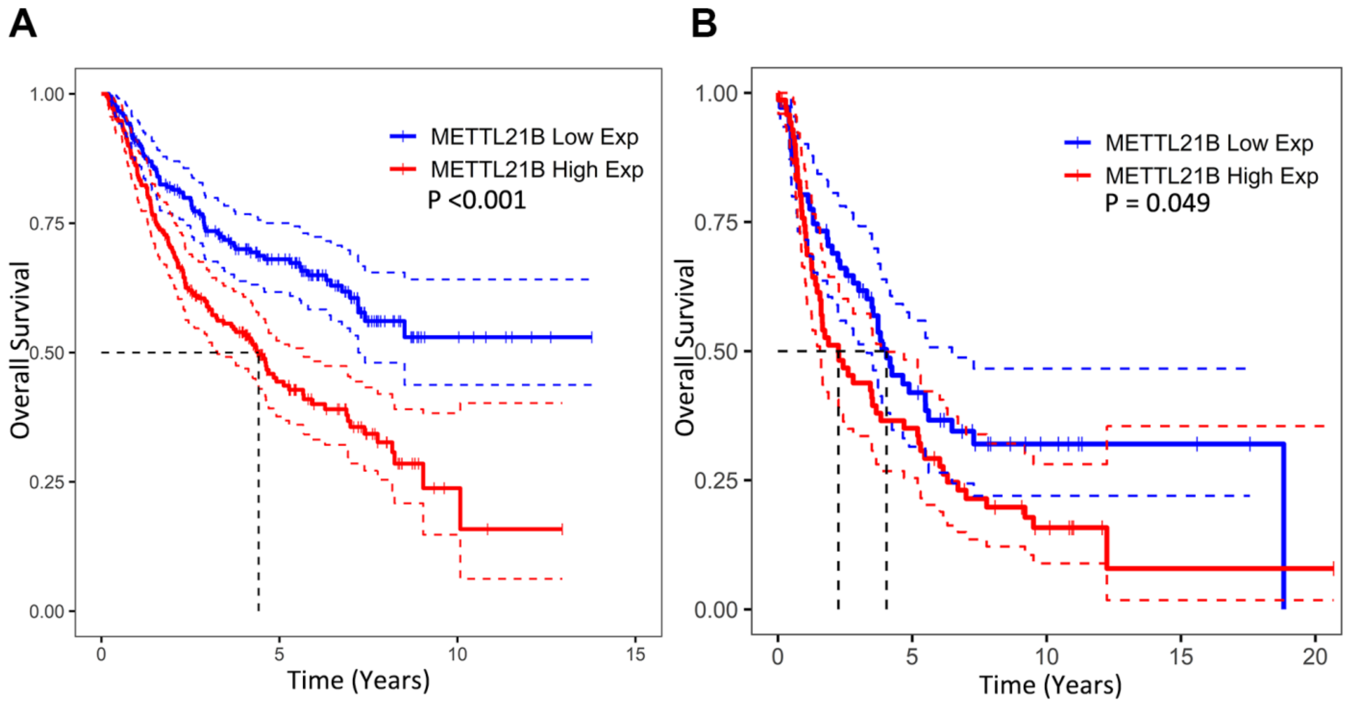

Supplementary Figure 2. The effect of METTL21B expression on prognosis of patients with LGG in CGGA database (**A**) and Rembrandt database (**B**).

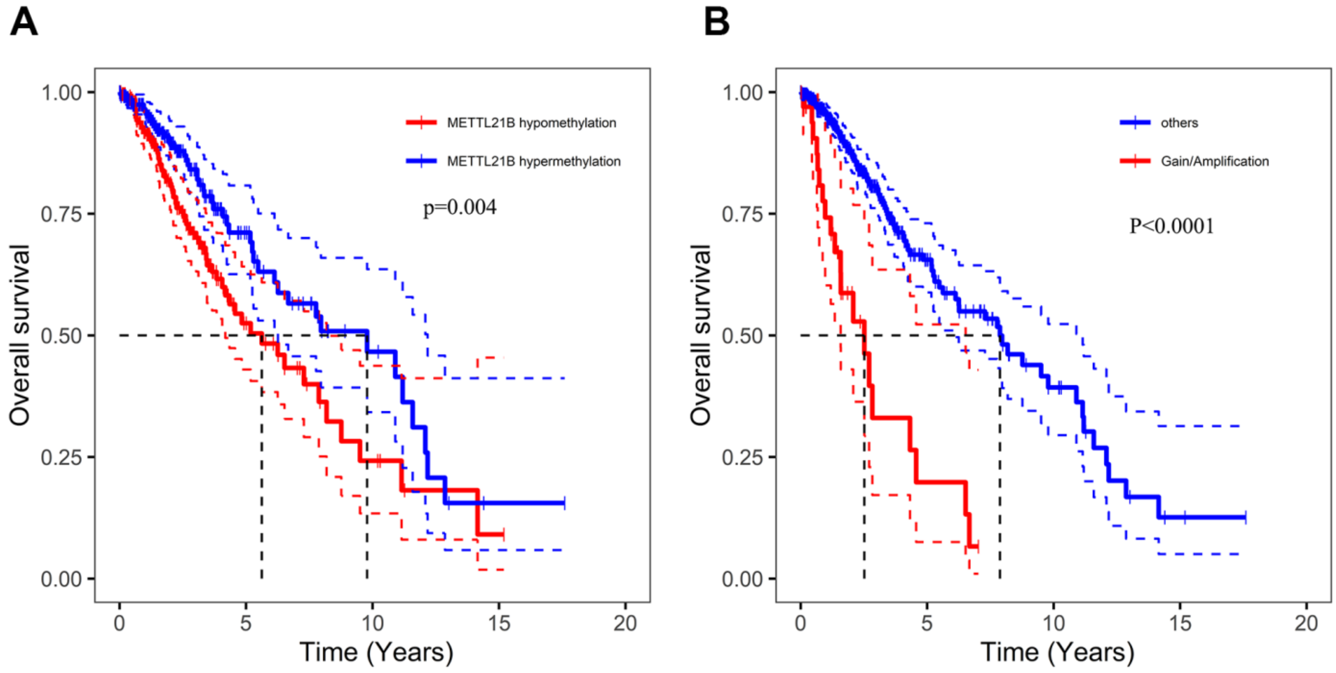

Supplementary Figure 3. The effect of METTL21B methylation (**A**) and CNV (**B**) on prognosis of patients with LGG.

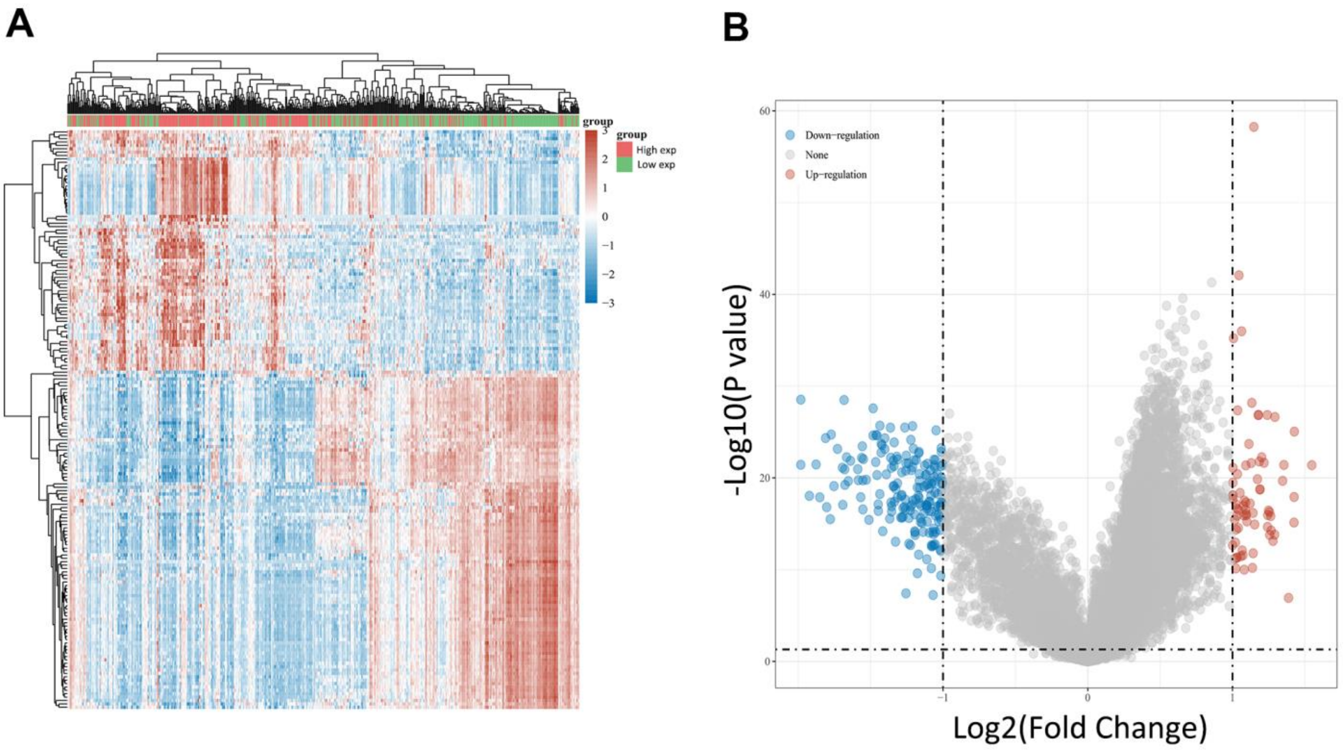

Supplementary Figure 4. Heatmap (**A**) and volcano plot (**B**) for DEGs.

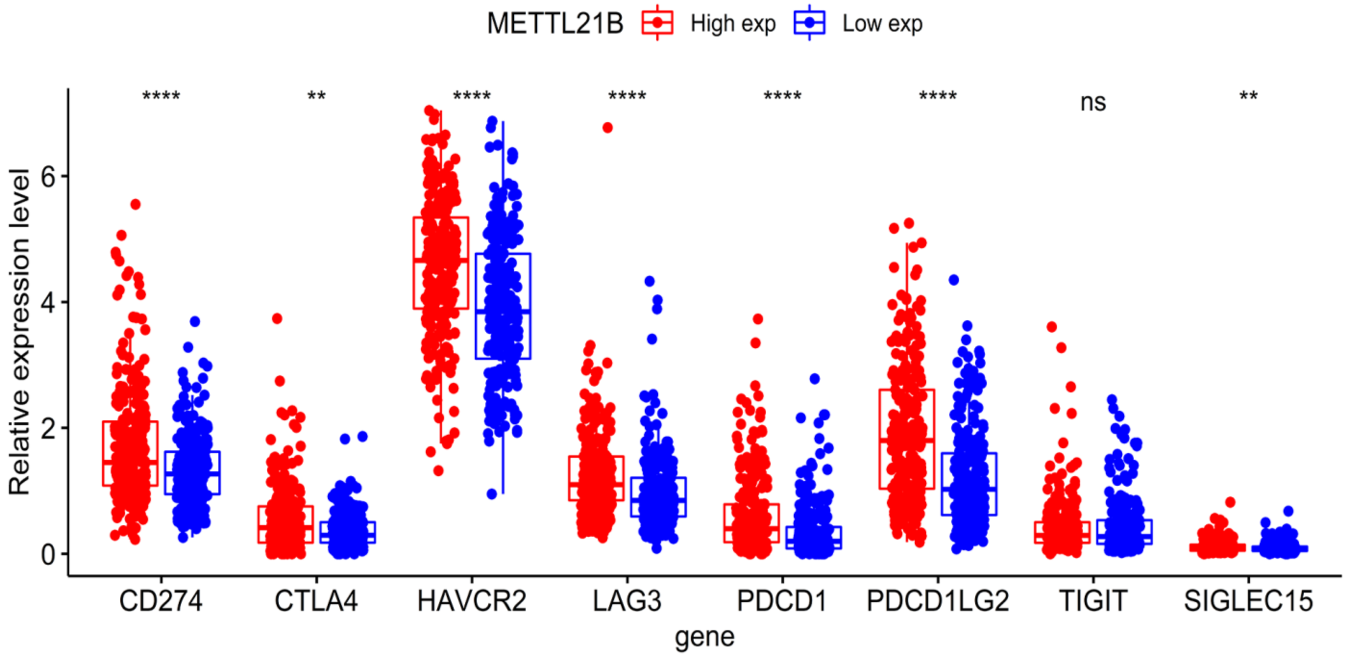

Supplementary Figure 5. The expression differences of 8 immune checkpoints between low and high METTL21B expression group. **P<0.01; ****P<0.0001; ns: no significance.
