# Supplementary material for: METTL21B is a prognostic biomarker and potential therapeutic target in low-grade gliomas
Source: Aging (Albany NY). 2021 Aug 26;13(16):20661–83. doi: 10.18632/aging.203454 (PMC8436898; doi:10.18632/aging.203454)
Supplement: Supplementary Table 1 [file aging-13-203454-s002.pdf]

SUPPLEMENTARY TABLE

Supplementary Table 1. The primers used for qPCR in this study.

Gene	Forward primer	Reverse primer
h- METTL21B	GTGGATTTCCGAGGCAAGAAGG	CAGGTCAGTGATGGTAACATCCC
h-GAPDH	AGGGGCCATCCACAGTCTTC	AGAAGGCTGGGGCTCATTTG
